# Effects of Sacubitril/Valsartan vs Valsartan in De Novo vs Acute on Chronic HFpEF and HFmrEF

**DOI:** 10.1016/j.jacadv.2024.100984

**Published:** 2024-05-14

**Authors:** Evan M. Murray, Derek Cyr, Marat Fudim, Jonathan H. Ward, Adrian F. Hernandez, Serge Lepage, David A. Morrow, Randall C. Starling, Kristin M. Williamson, Akshay S. Desai, Shelley Zieroth, Scott D. Solomon, Robert J. Mentz

**Affiliations:** a Department of Medicine, Duke University Hospital, Durham, North Carolina, USA; b Duke Clinical Research Institute, Durham, North Carolina, USA; c Novartis Pharmaceuticals Corporation, East Hanover, New Jersey, USA; d Department of Cardiology, Université de Sherbrooke, Sherbrooke, Quebec, Canada; e Cardiovascular Division, Department of Medicine, Brigham and Women’s Hospital, Harvard Medical School, Boston, Massachusetts, USA; f Department of Cardiovascular Medicine, Cleveland Clinic, Cleveland, Ohio, USA; g Section of Cardiology, Max Rady College of Medicine, University of Manitoba, Winnipeg, Manitoba, Canada

**Keywords:** chronic heart failure, de novo heart failure, heart failure with mid-range ejection fraction, heart failure with preserved ejection fraction, sacubitril/valsartan

## Abstract

**Background:**

Decompensated heart failure (HF) can be categorized as de novo or worsening of chronic HF. In PARAGLIDE-HF (Prospective comparison of ARNI with ARB Given following stabiLization In DEcompensated HFpEF), among patients with an ejection fraction >40% that stabilized after worsening HF, sacubitril/valsartan led to a significantly greater reduction in N-terminal pro-B-type natriuretic peptide (NT-proBNP) and was associated with clinical benefit compared to valsartan.

**Objectives:**

This prespecified analysis characterized patients with de novo vs worsening chronic HF in PARAGLIDE-HF and assessed the interaction between HF chronicity and the effect of sacubitril/valsartan.

**Methods:**

Patients were classified as de novo (first diagnosis of HF) or chronic (known HF prior to the index event). Time-averaged proportional change in NT-proBNP from baseline to weeks 4 and 8 was analyzed using an analysis of covariance model. A win ratio consisting of time to cardiovascular death, number and times of HF hospitalizations during follow-up, number and times of urgent HF visits during follow-up, and time-averaged proportional change in NT-proBNP was assessed for each group.

**Results:**

Of the 466 participants, 153 (33%) had de novo HF and 313 (67%) had chronic HF. De novo patients had lower rates of atrial fibrillation/flutter and lower creatinine. There was a nonsignificant reduction in NT-proBNP with sacubitril/valsartan vs valsartan for de novo (0.82; 95% CI: 0.62-1.07) and chronic HF (0.88; 95% CI: 0.73-1.07), interaction *P* = 0.66. The win ratio was nominally in favor of sacubitril/valsartan for both de novo (1.12; 95% CI: 0.70-1.58) and chronic HF (1.24; 95% CI: 0.89-1.71).

**Conclusions:**

There is no interaction between HF chronicity and the effect of sacubitril-valsartan.

Acute decompensated heart failure (HF) can be subclassified into 2 categories: worsening chronic HF and de novo HF.^1^ The distinction between these 2 entities has important implications for prognosis, responses to medical therapy, and the risk of future decompensation.^2^ In a previous analysis of patients with acute decompensated HF and an ejection fraction (EF) <40%, patients with de novo HF were younger, had fewer comorbidities, and experienced a lower cumulative incidence of cardiovascular (CV) death or HF rehospitalization, but still gained a similar benefit from sacubitril/valsartan as compared to enalapril with regard to reduction in N-terminal pro-B-type natriuretic peptide (NT-proBNP) at weeks 4 and 8.^3^

Sacubitril/valsartan is safe, well tolerated, and may lead to clinical benefit in patients with chronic stable HFpEF, particularly in patients with left ventricular ejection fraction (LVEF) on the lower end of the spectrum.^4^ The recently published PARAGLIDE-HF (Prospective comparison of ARNI with ARB Given following stabiLization In DEcompensated HFpEF) study extended these findings to patients with an EF >40% and a recent worsening HF event.^5^ The trial showed a greater time-averaged reduction in NT-proBNP with sacubitril/valsartan compared to valsartan, with a lower risk of worsening renal function in the sacubitril/valsartan arm but an increased incidence of symptomatic hypotension.

In the primary analysis of PARAGLIDE-HF, stratification by prior history of HF showed no significant interaction for the primary outcome of reduction in NT pro-BNP. However, a comparison of baseline demographics and an analysis of key secondary endpoints, including safety outcomes and clinical events, have not been performed. Furthermore, as the primary results suggested greater efficacy of sacubitril/valsartan among patients with an EF below normal (≤60%), subsequent analysis would benefit from highlighting this particular subgroup. Thus, given the importance of further characterizing patients with either de novo or worsening chronic HF, we undertook the current prespecified analysis to determine the relative effect of sacubitril/valsartan compared to valsartan in these important patient populations.

## Methods

### Trial design

The trial design and rationale for PARAGLIDE-HF have previously been reported.^6^ In brief, PARAGLIDE-HF was a multicenter, randomized, double-blind controlled trial comparing sacubitril/valsartan vs valsartan in patients with HF and an EF >40% following stabilization after a recent worsening heart failure (WHF) event. The primary endpoint was time-averaged proportional change in NT-proBNP from baseline to weeks 4 and 8. The ethics committee at each trial center approved the trial, and all patients provided written informed consent.

### Participants

The trial included 466 participants aged ≥18 years with a diagnosis of HF, an EF >40%, and an elevated NT-proBNP or B-type natriuretic peptide who were stabilized either during current hospitalization for HF or within 30 days of a WHF event (defined as HF hospitalization, emergency department visit, or out-of-hospital urgent HF visit, all requiring intravenous diuretics). Patients were defined as de novo HF if they did not carry a previous diagnosis of HF at the time of trial enrollment.

### Trial outcomes

The primary endpoint was the time-averaged proportional change in NT pro-BNP from baseline to weeks 4 and 8. Secondary endpoints were a win-ratio-based composite hierarchical outcome, cumulative number of composite HF events over time (CV death, HF hospitalizations, urgent HF visits), worsening renal function, and adverse events of special interest, as outlined below. Additionally, study drug dose levels were assessed for each group.

Given the larger demonstrated benefit of sacubitril/valsartan among patients with a below-normal EF (≤60%) in the overall trial, additional analyses among patients belonging to this subgroup were prespecified for all endpoints.

### Statistical methods

Baseline characteristics, including demographics, clinical features of HF, medical history, selected vitals and labs, and concomitant medications were reported for patients stratified by HF group, de novo vs chronic. Differences were assessed using the chi-squared test or Fisher’s exact test for categorical variables and the Wilcoxon rank-sum test or Student’s t-test for categorical variables.

For the primary endpoint of change in NT pro-BNP, the time-averaged proportional change from baseline on a natural logarithmic scale was analyzed using an analysis of covariance model using data from Weeks 4 and 8 with treatment arm, HF group (de novo vs chronic), in-hospital/out-of-hospital randomization status, gender, and baseline LVEF (≤ median, > median) as fixed effect factors, age and the logarithmic baseline NT-proBNP as covariates, and the interaction of treatment by HF group. The values from weeks 4 and 8 were averaged, and the change from baseline in log transformed NT-proBNP was calculated as follows: ln (average post dose value) – ln (baseline value). The original study was powered only for the primary endpoint (change in NT pro-BNP) and not for secondary endpoints, which are considered exploratory in the present work.

The secondary endpoint of the composite hierarchical outcome consisting of (in order of priority): 1) time to CV death; 2) number and times of HF hospitalizations during follow-up; 3) number and times of urgent HF visits during follow-up; and 4) time-averaged proportional change in NT-proBNP (from baseline to weeks 4 and 8) was analyzed, estimating the unmatched win ratio by comparing every participant in the sacubitril/valsartan arm to every participant in the valsartan arm to determine a winner (unmatched pairing method). A component was only used as a tiebreaker in the pairwise comparison between 2 subjects if the comparison of components with a higher priority resulted in a tie. The estimated win ratio (the total number of wins in the sacubitril/valsartan arm divided by the total number of wins in the valsartan arm) was calculated. A win ratio >1.0 was in favor of the sacubitril/valsartan arm. The analysis was performed separately within the de novo and chronic HF groups.

The cumulative number of composite events (CV death, HF hospitalizations, urgent HF visits) was calculated. The time to these recurrent events was analyzed using the semiparametric proportional rate model.^7^ A rate ratio (RR) <1 indicates an effect in favor of sacubitril/valsartan arm. The RR was estimated from the aforementioned proportional rates model with the HF group, treatment arm, and in-hospital/out-of-hospital randomization as fixed factors and the HF-by-treatment arm interaction. Additionally, the time-to-first composite event was analyzed using a Cox proportional hazards model with the HF group, treatment arm, the HF-by-treatment interaction, and in-hospital/out-of-hospital randomization as factors.

Incidence of a composite renal endpoint of: renal death, reaching end-stage renal disease (sustained estimated glomerular filtration rate [eGFR] <15 ml/min/m^2^, chronic dialysis, or renal transplant), or ≥50% decline in eGFR relative to baseline was reported using a negative binomial regression model with the count data as the dependent variable. HF group, treatment arm, and in-hospital/out-of-hospital randomization were included as fixed-effect factors, as well as the HF-by-treatment interaction and log (follow-up duration) as the off-set.

The incidence of adverse events of special interests: symptomatic hypotension, hyperkalemia (potassium > 5.5 mEq/L), and worsening renal function, defined as an increase in serum creatinine of ≥0.5 mg/dL and worsening of the eGFR by at least 25%, was analyzed using a logistic regression model with HF group, treatment arm, in-hospital/out-of-hospital randomization as fixed factors, as well as the HF-by-treatment arm interaction.

Study drug dose levels (ie, dose levels 1, 2, and 3, no treatment, and off treatment) were summarized by treatment arm in participants with de novo vs chronic HF. Data were presented as counts (percentages), and differences between treatment arms were assessed using the chi-squared test or Fisher’s exact test, as appropriate. The full statistical analysis plan is available upon request.

## Results

Of the 466 trial participants, 153 (33%) had de novo HF, and 313 (67%) had chronic HF. Baseline demographics were overall similar between the groups, with de novo patients having lower rates of atrial fibrillation or flutter and lower serum creatinine (Table 1).

**Table 1:** Baseline Characteristics of Randomized Participants According to Heart Failure Group

	De Novo Heart Failure (N = 153)	Chronic Heart Failure (N = 313)	*P* Value
Demographics			
Age (y)			0.209
N	153	313	
Median (Q1, Q3)	70 (60, 79)	72 (63, 79)	
Sex			0.495
Male	77/153 (50.3%)	147/313 (47.0%)	
Female	76/153 (49.7%)	166/313 (53.0%)	
Race			0.749
White	121/153 (79.1%)	231/313 (73.8%)	
Black or African American	29/153 (19.0%)	73/313 (23.3%)	
Asian	2/153 (1.3%)	4/313 (1.3%)	
Native Hawaiian or other Pacific Islander	0/153 (0.0%)	2/313 (0.6%)	
American Indian or Alaska Native	1/153 (0.7%)	3/313 (1.0%)	
Ethnicity			0.436
Hispanic or Latino	7/152 (4.6%)	20/312 (6.4%)	
Not Hispanic or Latino	145/152 (95.4%)	292/312 (93.6%)	
Clinical features of heart failure			
Ischemic etiology	N/A	82/310 (26.5%)	N/A
Prior HF hospitalization	N/A	182/313 (58.1%)	N/A
NYHA functional class			0.094
I	6/153 (3.9%)	11/310 (3.5%)	
II	78/153 (51.0%)	125/310 (40.3%)	
III	67/153 (43.8%)	162/310 (52.3%)	
IV	2/153 (1.3%)	12/310 (3.9%)	
Screening NT-proBNP (pg/mL) - local lab			0.254
N	83	178	
Median (Q1, Q3)	2,133 (1,589, 3,670)	1,959 (1,253, 3,827)	
Screening NT-proBNP (pg/mL) - central lab			0.051
N	148	302	
Median (Q1, Q3)	1,467 (705, 2,479)	1,658 (866, 3,145)	
Screening BNP (pg/mL) - local lab			0.129
N	70	131	
Median (Q1, Q3)	493 (300, 673)	515 (358, 877)	
LVEF, %			0.239
N	153	313	
Mean ± SD	54.9 ± 8.7	55.7 ± 7.7	
Median (Q1, Q3)	55 (47, 60)	55 (50, 60)	
Min, Max	41, 76	41, 80	
LVEF categories			0.032
41%-49%	46/153 (30.1%)	61/313 (19.5%)	
50%-60%	72/153 (47.1%)	178/313 (56.9%)	
>60%	35/153 (22.9%)	74/313 (23.6%)	
Medical history			
Hypertension	144/153 (94.1%)	303/313 (96.8%)	0.168
Diabetes mellitus	71/153 (46.4%)	155/313 (49.5%)	0.527
Prior atrial fibrillation/atrial flutter	70/153 (45.8%)	203/313 (64.9%)	<0.001
Stroke	11/152 (7.2%)	37/313 (11.8%)	0.127
Myocardial infarction	5/153 (3.3%)	22/313 (7.0%)	0.103
Examination and laboratory values			
Systolic blood pressure (mm Hg)			0.711
N	153	313	
Median (Q1, Q3)	127 (119, 145)	128 (116, 145)	
Heart rate (bpm)			1.000
N	153	313	
Median (Q1, Q3)	75 (65, 85)	73 (65, 88)	
BMI (kg/m^2^)			0.162
N	153	313	
Median (Q1, Q3)	32.7 (27.0, 39.5)	33.2 (27.7, 41.3)	
Serum creatinine (mg/dL)			<0.001
N	149	303	
Median (Q1, Q3)	1.1 (0.9, 1.4)	1.3 (1.0, 1.6)	
eGFR (mL/min/1.73 m^2^)			<0.001
N	148	302	
Median (Q1, Q3)	54.6 (40.7, 69.7)	47.0 (36.8, 58.9)	
Serum potassium (mmol/L)			0.061
N	148	300	
Median (Q1, Q3)	4.3 (4.1, 4.6)	4.3 (3.9, 4.5)	
Medications (prior and concomitant)			
Prior use of ACEI or ARB	82/153 (53.6%)	167/313 (53.4%)	0.961
ACEI or ARB at screening	117/153 (76.5%)	242/313 (77.3%)	0.838
MRA at randomization	37/153 (24.2%)	98/313 (31.3%)	0.111
Beta-blocker at randomization	113/153 (73.9%)	241/313 (77.0%)	0.456
SGLT2i at randomization	16/153 (10.5%)	40/313 (12.8%)	0.469
Diuretic agent at baseline	153/153 (100.0%)	312/313 (99.7%)	1.000
Other characteristics			
Randomization location			0.574
In-hospital	109/153 (71.2%)	215/313 (68.7%)	
Out-of-hospital	44/153 (28.8%)	98/313 (31.3%)	

ACEI = angiotensin converting enzyme inhibitor; ARB = angiotensin receptor blocker; BMI = body mass index; BNP = B-type natriuretic peptide; eGFR = estimated glomerular filtration rate; HF = heart failure; LVEF = left ventricular ejection fraction; MRA = mineralocorticoid receptor antagonist; NT-proBNP = N-terminal pro-brain natriuretic peptide; SGLT2i = sodium-glucose cotransporter-2 inhibitor.

**Figure 1:**
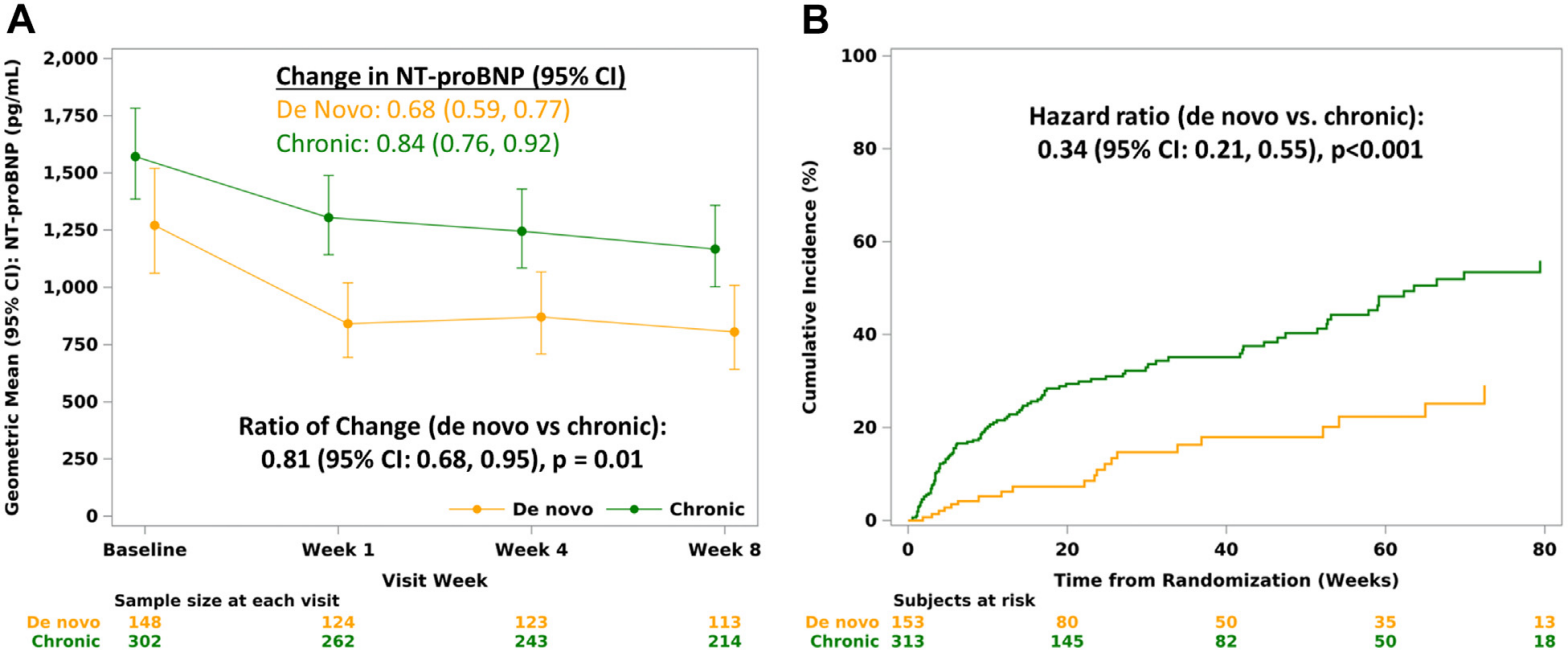
Differences in NT-proBNP and Composite Events for De Novo vs Chronic Heart Failure (A) Change in NT-proBNP from baseline through week 8: de novo vs chronic heart failure. Change in NT-proBNP from baseline through week 8. Ratio of change in NT-proBNP is presented as the ratio of geometric means between baseline value and the average of weeks 4 and 8 between the de novo and chronic HF groups. (B) Time to the first composite event (cardiovascular death, HF hospitalization, urgent HF visit) stratified by Heart Failure Group (de novo vs chronic). Secondary composite endpoint is analyzed using Cox proportional hazards regression with treatment, baseline heart failure group, and in-hospital/out-of-hospital randomization as fixed-effect factors. A hazard ratio <1.00 indicates an effect in favor of de novo heart failure group. HF = heart failure; NT-proBNP = N-terminal pro-brain natriuretic peptide.

When comparing all patients regardless of treatment group, those with chronic HF showed a higher baseline NT-proBNP, while those with de novo HF saw a larger time-averaged proportional change in NT-proBNP at weeks 4 and 8 (Figure 1A). The rate of composite clinical events of CV death, HF hospitalizations, and urgent HF visits was lower among those with de novo compared to chronic HF (HR 0.34 [95% CI: 0.21-0.55], *P* < 0.001) (Figure 1B).

**Figure 2:**
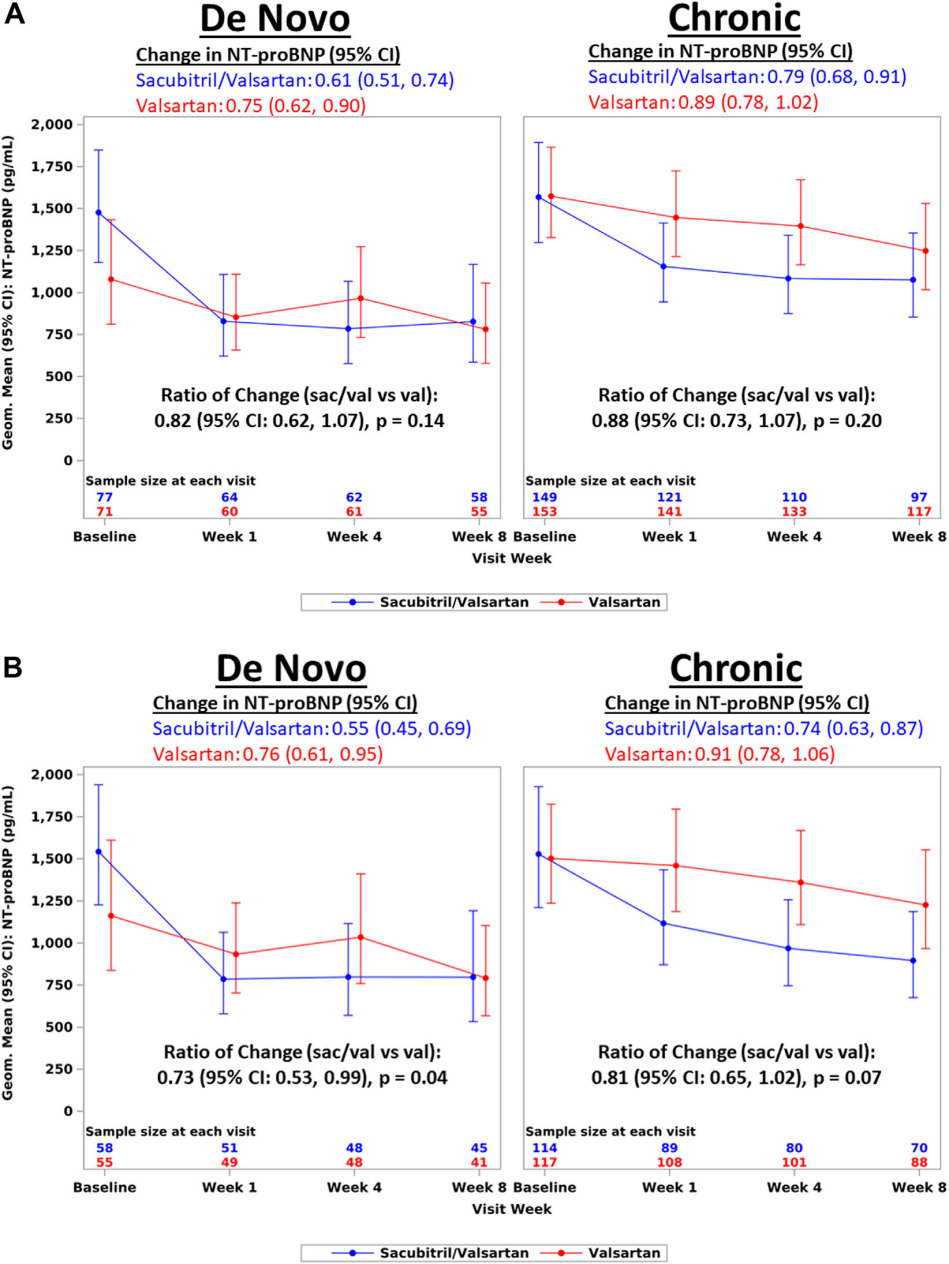
Change in NT-proBNP According to Treatment Arm by Baseline Heart Failure Group (De Novo vs. Chronic) (A) Change in NT-proBNP according to treatment arm by baseline heart failure group. Change in NT-proBNP is shown graphically as geometric mean at baseline and each subsequent follow-up visit. Numeric change is presented as ratio of geometric mean at baseline compared to average of geometric means at weeks 4 and 8. Ratio of change is presented as ratio of change from baseline to weeks 4 and 8 in sacubitril/valsartan arm compared to valsartan arm. *P* interaction = 0.66. (B) Change in NT-proBNP from baseline according to treatment arm by baseline Heart Failure Group, subgroup With EF ≤60%. Change in NT-proBNP is shown graphically as geometric mean at baseline and each subsequent follow-up visit. Numeric change is presented as ratio of geometric mean at baseline compared to average of geometric means at weeks 4 and 8. Ratio of change presented as ratio of change from baseline to weeks 4 and 8 in sacubitril/valsartan arm compared to valsartan arm. *P* interaction = 0.56. EF = ejection fraction; NT-proBNP = N-terminal pro-brain natriuretic peptide.

The time-averaged proportional change in NT-proBNP was similar for sacubitril/valsartan in both de novo and chronic HF (Central Illustration). There was no interaction by HF chronicity (*P* interaction = 0.66) (Figure 2A). Among the group of patients with LVEF ≤60%, a similar effect was seen for both de novo and chronic HF (*P* interaction = 0.56) (Figure 2B).

**Central Illustration:**
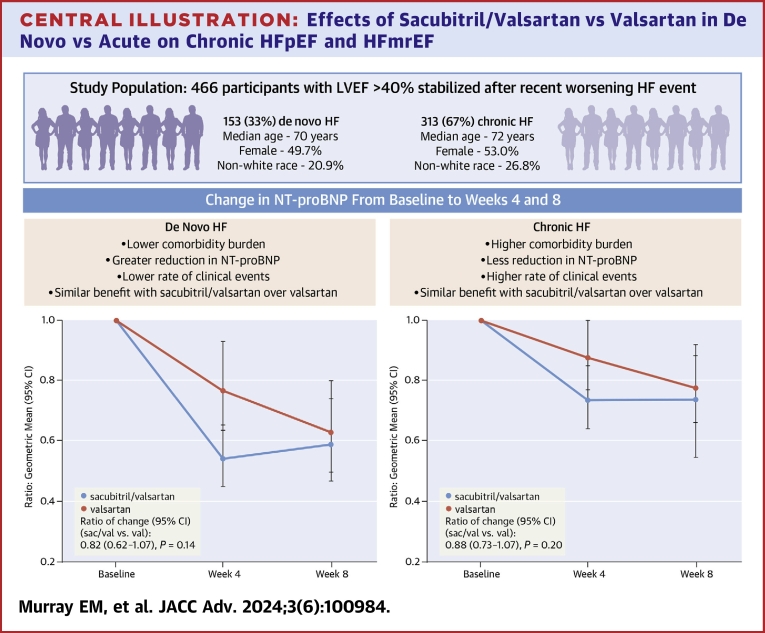
**Effects of Sacubitril/Valsartan vs Valsartan in De Novo vs Acute on Chronic HFpEF and HFmrEF** Time averaged proportional change in NT-proBNP as ratio of geometric means from baseline to weeks 4 and 8 for sacubitril/valsartan vs valsartan in de novo and chronic heart failure. *P* interaction = 0.66. HF = heart failure; HFpEF = heart failure with preserved ejection fraction; HFmrEF = heart failure with mid-range ejection fraction; LVEF = left ventricular ejection fraction; NT-proBNP = N-terminal pro-brain natriuretic peptide.

Secondary outcomes are presented in Table 2. The composite hierarchical outcome by win ratio favored sacubitril/valsartan among both de novo and chronic HF, although neither group met statistical significance. Among patients with EF ≤60%, the win ratio again showed a more pronounced effect of sacubitril/valsartan in both groups.

**Table 2:** Secondary Outcomes

		Sacubitril/Valsartan		Valsartan		Sacubitril/Valsartan vs Valsartan		
Hierarchical Composite of CV Death, HF Hospitalizations, Urgent HF Visits, and Change in NT-proBNP								
	Heart Failure Group	n	% Wins	n	% Wins	Win Ratio (95% CI)	*P* Value	*P* Interaction
All participants	De novo	78	32.4%	75	28.8%	1.12 (0.70–1.91)	0.61	0.72
	Chronic	155	39.4%	158	31.8%	1.24 (0.89–1.71)	0.15	
LVEF ≤60%	De novo	59	35.9%	59	24.2%	1.48 (0.88–2.80)	0.14	1.00
	Chronic	119	39.5%	120	26.7%	1.48 (1.01–2.20)	0.03	

Recurrent CV Composite Events of HF Hospitalizations, Urgent HF Visits, and CV Death								
	Heart Failure Group	n	EAR (95% CI)^a^	n	EAR (95% CI)^a^	Rate Ratio (95% CI)	*P* Value	*P* Interaction
All participants	De novo	78	29.6 (15.8–50.7)	75	28.5 (15.9–47.0)	1.00 (0.41–2.46)	0.99	0.63
	Chronic	155	77.8 (61.8–96.7)	158	101.1 (82.4–122.7)	0.78 (0.52–1.19)	0.25	
LVEF ≤60%	De novo	59	21.5 (8.6–44.3)	59	33.8 (18.5–56.7)	0.59 (0.21–1.72)	0.34	0.97
	Chronic	119	56.5 (40.9–76.1)	120	97.2 (76.9–121.1)	0.61 (0.35–1.06)	0.08	

Renal Composite Endpoint of Renal Death, ESRD, or >50% Decline in eGFR								
	Heart Failure Group	n	Estimated Event Rate^b^ (95% CI)	n	Estimated Event Rate (95% CI)	Rate Ratio (95% CI)	*P* Value	*P* Interaction
All participants	De novo	78	0.36 (0.12–1.11)	75	0.45 (0.13–1.50)	0.81 (0.16–4.15)	0.80	0.83
	Chronic	155	1.06 (0.44–2.56)	158	1.62 (0.78–3.38)	0.65 (0.22–1.95)	0.44	
LVEF ≤60%	De novo	59	0.54 (0.16–1.85)	59	0.47 (0.11–2.00)	1.16 (0.18–7.38)	0.87	0.58
	Chronic	119	0.69 (0.23–2.13)	120	1.15 (0.49–2.67)	0.60 (0.16–2.31)	0.46	

Symptomatic Hypotension								
	Heart Failure Group	n	Number of Subjects (%)	n	Number of Subjects (%)	OR (95% CI)	*P* Value	*P* Interaction
All participants	De novo	78	14 (18.0)	75	10 (13.3)	1.42 (0.59–3.44)	0.43	0.59
	Chronic	155	42 (27.1)	158	26 (16.5)	1.89 (1.09–3.27)	0.02	
LVEF ≤60%	De novo	59	9 (15.3)	59	7 (11.9)	1.34 (0.46–3.88)	0.59	0.98
	Chronic	119	28 (23.5)	120	22 (18.3)	1.36 (0.73–2.56)	0.33	
Hyperkalemia^c^								
All participants	De novo	78	15 (19.2)	75	13 (17.3)	1.12 (0.49–2.54)	0.80	0.88
	Chronic	155	30 (19.4)	158	30 (19.0)	1.03 (0.59–1.82)	0.91	
LVEF ≤60%	De novo	59	11 (18.6)	59	10 (17.0)	1.12 (0.43–2.87)	0.82	0.88
	Chronic	119	22 (18.5)	120	22 (18.3)	1.02 (0.53–1.96)	0.95	
Worsening renal function^d^								
All participants	De novo	78	9 (11.5)	75	17 (22.7)	0.45 (0.18–1.08)	0.07	0.42
	Chronic	155	41 (26.5)	158	55 (34.8)	0.67 (0.41–1.09)	0.11	
LVEF ≤60%	De novo	59	9 (15.3)	59	12 (20.3)	0.71 (0.27–1.83)	0.47	0.60
	Chronic	119	27 (22.7)	120	43 (35.8)	0.52 (0.30–0.93)	0.03	

CV = cardiovascular; eGFR = estimated glomerular filtration rate; ESRD = End Stage Renal Disease; HF = heart failure; LVEF = left ventricular ejection fraction; NT-proBNP = N-terminal pro-brain natriuretic peptide.

a EAR: exposure adjusted rate per 100 subject-years.

b Calculated by a negative binomial (NB) regression model, adjusted for baseline heart failure group, treatment arm, in-hospital/out-of-hospital randomized, and the interaction of baseline heart failure group by treatment arm. Log (follow-up duration, study exposure) is the offset variable. Events that occurred in the randomized double-blind treatment period are included in the analysis.

c Defined as potassium >5.5 mEq/L.

d Increase in serum creatinine of >0.5 mg/dl AND worsening of eGFR by at least 25%.

There was no significant difference in the effects of sacubitril/valsartan in de novo or chronic HF on the secondary composite outcome of CV death, HF hospitalizations, or urgent HF visits. However, those with chronic HF and EF ≤60% saw a significantly lower rate of HF hospitalizations with sacubitril/valsartan (RR: 0.55; 95% CI 0.32-0.95; *P* = 0.03). This effect was not seen in de novo HF patients with EF ≤60% (RR: 1.22; 95% CI: 0.33-4.57; *P* = 0.77). The interaction term was not significant for this effect (*P* interaction = 0.28).

**Figure 3:**
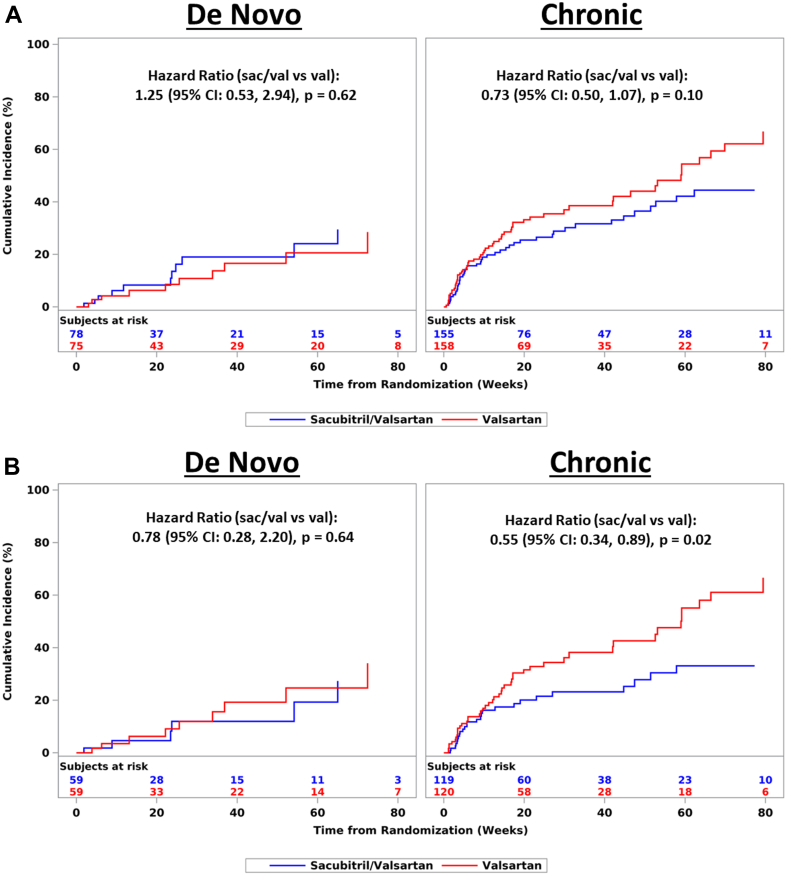
Time to First Composite Event According to Treatment Arm by Baseline Heart Failure Group (De Novo vs. Chronic) (A) Time to first composite event (CV death, HF hospitalization, urgent HF visit) stratified by treatment within heart failure groups. Secondary composite endpoint is analyzed using Cox proportional hazards regression with treatment, baseline heart failure group, in-hospital/out-of-hospital randomization, and the interaction of treatment by baseline heart failure group. A hazard ratio <1.00 indicates an effect in favor of sacubitril/valsartan. *P* interaction = 0.27. (B) Time to first composite event (CV death, HF hospitalization, urgent HF visit) stratified by treatment within Heart Failure Groups, subgroup with EF ≤60%. Secondary composite endpoint is analyzed using Cox proportional hazards regression with treatment, baseline heart failure group, in-hospital/out-of-hospital randomization, and the interaction of treatment by baseline heart failure group. A hazard ratio <1.00 indicates an effect in favor of sacubitril/valsartan. *P* interaction = 0.56. CV = cardiovascular; EF = ejection fraction; HF = heart failure.

Time-to-first recurrent event (CV death, HF hospitalization, or urgent HF visit) for sacubitril/valsartan vs valsartan did not significantly differ between de novo and chronic HF (Figure 3A). Among the EF ≤60% subgroup, the hazard ratio favored sacubitril/valsartan in the chronic group (HR: 0.55; 95% CI: 0.34-0.89; *P* = 0.02) but not the de novo group; however, the interaction term was not significant (*P* = 0.56) (Figure 3B).

There were no significant differences in the effect of sacubitril/valsartan on the composite renal endpoint of renal death, End Stage Renal Disease, or >50% decline in eGFR. Rates of symptomatic hypotension were significantly higher with sacubitril/valsartan in the chronic HF group, while the de novo group saw nominally higher rates of hypotension that did not meet statistical significance. For the endpoint of worsening renal function (increase in creatinine >0.5 and worsening of eGFR by >25%), the point estimate showed lower rates with sacubitril/valsartan in all groups, but only a significant reduction with sacubitril/valsartan in those with chronic HF and EF ≤60%. There were no differences in drug dose level achieved among either group (Supplemental Tables 1 and 2).

## Discussion

Among patients with EF >40% stabilized after a recent WHF event, both those with de novo and worsening chronic HF saw nominally larger reductions in NT-proBNP with sacubitril/valsartan compared to valsartan, without interaction by HF chronicity. These data suggest that sacubitril/valsartan may provide a similar effect in both de novo and worsening chronic HF patients in reducing natriuretic peptides.

The effect of sacubitril/valsartan seen in the overall trial population^5^ appeared similar among both de novo and chronic HF patients. Although the larger reduction of NT-proBNP with sacubitril/valsartan did not meet statistical significance in either de novo or chronic HF individual subgroups, this can likely be explained by the loss of statistical power when accounting for the smaller sample size. Importantly, there was no significant interaction by HF chronicity, indicating that sacubitril/valsartan’s effect on reduction in natriuretic peptides did not appear to be modified by de novo vs chronic HF group.

Several previous studies have described differences in de novo and worsening chronic HF patients.^1^^,^^8^^,^^9^ De novo patients are typically younger with a lower burden of comorbidities than their counterparts with chronic HF. In this study, we also found a higher rate of prior atrial fibrillation or flutter and a higher serum creatinine in the chronic HF group, despite similar age, vital signs, and medication use at baseline. This higher burden of comorbidities likely played a role in the worse clinical outcomes among this group.

The risk of cardiovascular events has been consistently demonstrated in the literature to be higher among chronic patients compared to de novo patients.^10^^,^^11^ This was again shown in the data from PARAGLIDE-HF presented in this study, where chronic HF patients saw significantly higher rates of clinical events and higher NT-proBNP in follow-up compared to de novo patients. This lower event rate among de novo patients, combined with the underpowering of this study for clinical endpoints, may explain the lack of statistically significant clinical benefit with sacubitril/valsartan seen by the de novo group. Furthermore, when looking specifically at the subset of patients with chronic HF and an EF ≤60%, which likely represent the highest-risk subgroup, the win ratio significantly favored sacubitril/valsartan, and there was a significantly lower rate of HF hospitalizations and clinical events. The lack of significant statistical interaction for these endpoints by HF chronicity, however, shows that these differential effects may be driven by higher event rates in this group rather than a difference in efficacy of sacubitril/valsartan among the de novo vs chronic phenotypes.

Safety endpoints including changes in renal function and rates of symptomatic hypotension were also important considerations in this trial. In the overall trial results, sacubitril/valsartan had higher rates of symptomatic hypotension compared to valsartan. This effect was seen among chronic HF patients but not in de novo patients. There were also lower rates of worsening renal function with sacubitril/valsartan in the overall trial results, an effect that persisted in the chronic HF group with LVEF ≤60% in this analysis. These findings suggest that chronic HF patients are at high risk of medication side effects and should be monitored closely for adverse events; however, they still stand to benefit from the drug, especially with regard to preventing renal dysfunction.

This study is the first to look at the effect of sacubitril/valsartan among de novo vs chronic HF patients in patients with a mildly reduced or preserved EF (>40%). A previous analysis of patients from PIONEER-HF similarly showed that among patients with an EF ≤40%, sacubitril/valsartan was safe, well-tolerated, and led to a greater reduction in NT-proBNP compared to enalapril, regardless of prior history of HF.^3^ This study extends those findings to patients across the range of EFs, again suggesting similar outcomes of sacubitril/valsartan regardless of chronicity of HF with respect to lowering natriuretic peptide levels.

### Below-normal EF

This study included an additional analysis of patients with a “below normal” (≤60%) EF. The rationale for including this is because this appears to be the population for which sacubitril/valsartan has the largest benefit with respect to reducing clinical events^12^ and specific United States Food and Drug Administration labeling for use of sacubitril/valsartan for patients in this population.^13^ Likewise, in the primary analysis of PARAGLIDE-HF, the win ratio for clinical events significantly favored sacubitril/valsartan only in the EF ≤60% population, highlighting the special utility in this key demographic.

In this analysis, we again see potentially stronger evidence of benefit with sacubitril/valsartan among the below-normal EF patient population. For the primary endpoint in change in NT-proBNP, the point estimate for reduction with sacubitril/valsartan suggests a greater reduction among EF ≤60% group for both de novo and chronic HF. The win ratio has higher estimates in favor of sacubitril/valsartan for the EF ≤60% group for both de novo and chronic HF, meeting significance only for the chronic group. Although it is difficult to directly compare win ratios from different trials, the magnitude of the win ratio seen in the EF ≤60% group for both de novo (1.45) and chronic (1.45) HF is similar to that seen for empagliflozin in EMPULSE^14^ (1.36) and greater than that of ferric carboxymaltose in HEART-FID^15^ (1.10). However, in all participants regardless of EF, the point estimate for the win ratio in favor of sacubitril/valsartan is numerically smaller than that in the EF ≤60% group, 1.12 for de novo and 1.24 for chronic, suggesting that the efficacy of sacubitril-valsartan in this population may depend on careful patient selection, ie, those with below-normal EF.

These findings suggest that once removing the “noise” of the population least likely to benefit (EF >60%), the beneficial effect of sacubitril/valsartan appears to be even stronger, regardless of HF chronicity. Direct comparison between EF ≤60% and >60% was not within the scope of this paper, and analysis of the >60% group was not performed due to small sample size in this group. Although a small sample size may limit the power of this analysis, future studies could specifically recruit patients with a below normal EF to provide further evidence.

### Study limitations

This study is a prespecified subgroup analysis but is nonetheless hypothesis-generating, and thus causations cannot be inferred from the associations presented here. Furthermore, the smaller sample size created by subgroup analysis, in addition to a low event rate, may result in limited power to detect differences between groups. Randomization was not stratified by HF chronicity, and there may be residual unmeasured confounding that affects the outcomes of this study.

## Conclusions

Among patients with an EF >40% with a recent WHF event, those with de novo HF had a lower risk of clinical events but a similarly large reduction in NT-proBNP with sacubitril/valsartan vs valsartan when compared to patients with *chronic* HF.

Perspectives **COMPETENCY IN MEDICAL KNOWLEDGE:** Sacubitril/valsartan is safe and well tolerated among patients with an EF >40% and either de novo or worsening chronic HF. The beneficial effect of sacubitril/valsartan does not appear to be significantly different between patients with either de novo or chronic HF.

**TRANSLATIONAL OUTLOOK:** De novo and worsening chronic HF are 2 distinct patient populations. Both appear to benefit from sacubitril/valsartan. Future studies should identify and investigate tailored approaches to meeting the different needs of these distinct groups.

## Funding support and author disclosures

PARAGLIDE-HF was funded by Novartis Pharmaceuticals Corporation. Dr Mentz has received research support and/or honoraria from Novartis, Abbott, American Regent, Amgen, AstraZeneca, Bayer, Boehringer Ingelheim, Boston Scientific, Cytokinetics, Fast BioMedical, Gilead, Innolife, Eli Lilly, Medtronic, Medable, Merck, Novo Nordisk, Pharmacosmos, Relypsa, Respicardia, Roche, Sanofi, Vifor, Windtree Therapeutics, and Zoll. Drs Ward and Williamson are employees of Novartis. Dr Hernandez has received research grants from American Regent, Amgen, AstraZeneca, Bayer, Bayer, Boehringer Ingelheim, Cytokinetics, Merck, Novartis, Somologic, and Verily, and has served as a consultant for Amgen, AstraZeneca, Bayer, Bristol Myers Squibb, Boehringer Ingelheim, Boston Scientific, Cytokinetics, Merck, Novartis, and Novo Nordisk. Dr Morrow is a member of the TIMI Study Group, which has received institutional research grant support through Brigham and Women’s Hospital from Abbott, Abiomed, Amgen, Anthos Therapeutics, ARCA Biopharma, Inc, AstraZeneca, Bayer HealthCare Pharmaceuticals, Inc, Daiichi-Sankyo, Eisai, Intarcia, Ionis Pharmaceuticals, Inc, Janssen Research and Development, LLC, Merck, Novartis, Pfizer, Quark Pharmaceuticals, Regeneron Pharmaceuticals, Inc, Roche, Siemens Healthcare Diagnostics, Inc, Softcell Medical Limited, and Zora Biosciences, and has received consulting fees from Abbott Laboratories, ARCA Biopharma, Inflammatix, Merck and Co, Novartis, and Roche Diagnostics. Dr Starling serves on the steering committee for the PARAGLIDE trial sponsored by Novartis. Dr Desai has received research grants (to Brigham and Women’s Hospital) from Abbott, Alnylam, AstraZeneca, Bayer, and Novartis; and has received personal consulting fees from Abbott, Alnylam, AstraZeneca, Avidity Biopharma, Axon Therapeutics, Bayer, Biofourmis, Cytokinetics, GlaxoSmithKline, Medpace, Merck, New Amsterdam, Novartis, Parexel, Regeneron, River2Renal, Roche, Verily, and Veristat. Dr Zieroth has received research grant support, served on advisory boards for, or has had speaker engagements with Abbott, Akcea AstraZeneca, Amgen, Alnylam, Bayer, Bristol Myers Squibb, Boehringer Ingelheim, Eli Lilly, GlaxoSmithKline, Janssen, Merck, Novartis, Novo Nordisk, Otsuka, Pfizer, Roche, Servier, and Vifor Pharma; and serves on a clinical trial committee or as a national lead for studies sponsored by AstraZeneca, Bayer, Boehringer Ingelheim, Merck, Novartis, and Pfizer. Dr Solomon has received research grants from Alnylam, AstraZeneca, Bellerophon, Bayer, Bristol Myers Squibb, Cytokinetics, Eidos, GlaxoSmithKline, Ionis, Lilly, MyoKardia, National Institutes of Health/National Heart, Lung, and Blood Institute, Novartis, Novo Nordisk, Respicardia, Sanofi Pasteur, Theracos, Actelion, Amgen, Bellerophon, Celladon, Gilead, Mesoblast, Neurotronik, and US2; and has consulted for Abbott, Action, Akros, Alnylam, Amgen, Arena, AstraZeneca, Bayer, Boehringer Ingelheim, Bristol Myers Squibb, Cardior, Cardurion, Corvia, Cytokinetics, Daiichi-Sankyo, GlaxoSmithKline, Lilly, Merck, Myokardia, Novartis, Roche, Theracos, Quantum Genomics, Cardurion, Janssen, Cardiac Dimensions, Tenaya, Sanofi-Pasteur, Dinaqor, Tremeau, CellProThera, Moderna, American Regent, Sarepta, Lexicon, Anacardio, Akros, and Valo. All other authors have reported that they have no relationships relevant to the contents of this paper to disclose.
