# Supplementary material for: Effects of Sacubitril/Valsartan vs Valsartan in De Novo vs Acute on Chronic HFpEF and HFmrEF
Source: JACC Adv. 2024 May 14;3(6):100984. doi: 10.1016/j.jacadv.2024.100984 (PMC11198033; doi:10.1016/j.jacadv.2024.100984)
Supplement: Supplemental Tables 1 and 2 [file mmc1.docx]

**Supplemental Table 1: Baseline Characteristics of Randomized Participants According to Heart Failure Group and Treatment Arm**

|  | ***De novo* Heart Failure (N=153)** | |  | **Chronic Heart Failure (N=313)** | |
| --- | --- | --- | --- | --- | --- |
| **Characteristic** | **Sacubitril/Valsartan (N=78)** | **Valsartan (N=75)** |  | **Sacubitril/Valsartan (N=155)** | **Valsartan (N=158)** |
| **Demographics** |  |  |  |  |  |
| Age (yrs.) |  |  |  |  |  |
| N | 78 | 75 |  | 155 | 158 |
| Median (Q1, Q3) | 72 (60, 79) | 68 (59, 77) |  | 70 (62, 78) | 74 (63, 79) |
|  |  |  |  |  |  |
| Gender |  |  |  |  |  |
| Male | 35/78 (44.9%) | 42/75 (56.0%) |  | 77/155 (49.7%) | 70/158 (44.3%) |
| Female | 43/78 (55.1%) | 33/75 (44.0%) |  | 78/155 (50.3%) | 88/158 (55.7%) |
|  |  |  |  |  |  |
| Race |  |  |  |  |  |
| White | 62/78 (79.5%) | 59/75 (78.7%) |  | 114/155 (73.5%) | 117/158 (74.1%) |
| Black or African American | 14/78 (17.9%) | 15/75 (20.0%) |  | 36/155 (23.2%) | 37/158 (23.4%) |
| Asian | 1/78 (1.3%) | 1/75 (1.3%) |  | 2/155 (1.3%) | 2/158 (1.3%) |
| Native Hawaiian or Other Pacific Islander | 1/78 (1.3%) | 0/75 (0.0%) |  | 1/155 (0.6%) | 1/158 (0.6%) |
| American Indian or Alaska Native |  |  |  | 2/155 (1.3%) | 1/158 (0.6%) |
|  |  |  |  |  |  |
| Ethnicity |  |  |  |  |  |
| Hispanic or Latino | 4/78 (5.1%) | 3/74 (4.1%) |  | 14/155 (9.0%) | 6/157 (3.8%) |
| Not Hispanic or Latino | 74/78 (94.9%) | 71/74 (95.9%) |  | 141/155 (91.0%) | 151/157 (96.2%) |
|  |  |  |  |  |  |
| **Clinical Features of Heart Failure** |  |  |  |  |  |
| Ischemic Etiology | N/A | N/A |  | 44/153 (28.8%) | 38/157 (24.2%) |
|  |  |  |  |  |  |
| Prior HF Hospitalization | N/A | N/A |  | 84/155 (54.2%) | 98/158 (62.0%) |
|  |  |  |  |  |  |
| NYHA Class |  |  |  |  |  |
| Class I | 3/78 (3.8%) | 3/75 (4.0%) |  | 5/153 (3.3%) | 6/157 (3.8%) |
| Class II | 44/78 (56.4%) | 34/75 (45.3%) |  | 58/153 (37.9%) | 67/157 (42.7%) |
| Class III | 31/78 (39.7%) | 36/75 (48.0%) |  | 86/153 (56.2%) | 76/157 (48.4%) |
| Class IV | 0/78 (0.0%) | 2/75 (2.7%) |  | 4/153 (2.6%) | 8/157 (5.1%) |
|  |  |  |  |  |  |
| Screening NT-proBNP (pg/mL) - Local Lab |  |  |  |  |  |
| N | 39 | 44 |  | 95 | 83 |
| Median (Q1, Q3) | 2,428 (1,681, 4,370) | 2,081 (1,406, 3,505) |  | 2,199 (1,315, 3,813) | 1,740 (1,163, 3,990) |
|  |  |  |  |  |  |
| Screening NT-proBNP (pg/mL) - Central Lab |  |  |  |  |  |
| N | 77 | 71 |  | 149 | 153 |
| Median (Q1, Q3) | 1,523 (725, 2,808) | 1,231 (492, 2,276) |  | 1,650 (969, 3,068) | 1,682 (808, 3,213) |
|  |  |  |  |  |  |
| Screening BNP (pg/mL) - Local Lab |  |  |  |  |  |
| N | 38 | 32 |  | 58 | 73 |
| Median (Q1, Q3) | 491 (296, 653) | 515 (324, 916) |  | 493 (319, 871) | 561 (397, 877) |
|  |  |  |  |  |  |
| LVEF % |  |  |  |  |  |
| N | 78 | 75 |  | 155 | 158 |
| Mean (SD) | 55.0 (8.8) | 54.9 (8.8) |  | 55.3 (7.7) | 56.0 (7.7) |
| Median (Q1, Q3) | 55 (47, 60) | 55 (46, 60) |  | 55 (50, 60) | 55 (50, 60) |
| Min, Max | 41, 75 | 41, 76 |  | 41, 75 | 42, 80 |
|  |  |  |  |  |  |
| LVEF Categories |  |  |  |  |  |
| 41%-49% | 23/78 (29.5%) | 23/75 (30.7%) |  | 33/155 (21.3%) | 28/158 (17.7%) |
| 50%-60% | 36/78 (46.2%) | 36/75 (48.0%) |  | 86/155 (55.5%) | 92/158 (58.2%) |
| >60% | 19/78 (24.4%) | 16/75 (21.3%) |  | 36/155 (23.2%) | 38/158 (24.1%) |
|  |  |  |  |  |  |
| **Medical History** |  |  |  |  |  |
| Hypertension | 76/78 (97.4%) | 68/75 (90.7%) |  | 152/155 (98.1%) | 151/158 (95.6%) |
|  |  |  |  |  |  |
| Diabetes Mellitus | 35/78 (44.9%) | 36/75 (48.0%) |  | 72/155 (46.5%) | 83/158 (52.5%) |
|  |  |  |  |  |  |
| Prior Atrial Fibrillation/Atrial Flutter | 41/78 (52.6%) | 29/75 (38.7%) |  | 99/155 (63.9%) | 104/158 (65.8%) |
|  |  |  |  |  |  |
| Ongoing Atrial Fibrillation/Atrial Flutter | 34/35 (97.1%) | 28/28 (100.0%) |  | 86/89 (96.6%) | 92/94 (97.9%) |
|  |  |  |  |  |  |
| Stroke | 5/77 (6.5%) | 6/75 (8.0%) |  | 20/155 (12.9%) | 17/158 (10.8%) |
|  |  |  |  |  |  |
| Myocardial Infarction | 3/78 (3.8%) | 2/75 (2.7%) |  | 9/155 (5.8%) | 13/158 (8.2%) |
|  |  |  |  |  |  |
| **Examination and Laboratory Values** |  |  |  |  |  |
| Systolic Blood Pressure (mmHg) |  |  |  |  |  |
| N | 78 | 75 |  | 155 | 158 |
| Median (Q1, Q3) | 126 (119, 145) | 128 (120, 145) |  | 128 (116, 142) | 129 (117, 147) |
|  |  |  |  |  |  |
| Heart Rate (bpm) |  |  |  |  |  |
| N | 78 | 75 |  | 155 | 158 |
| Median (Q1, Q3) | 75 (63, 86) | 74 (66, 85) |  | 75 (66, 92) | 71 (64, 85) |
|  |  |  |  |  |  |
| BMI (kg/m^2^) |  |  |  |  |  |
| N | 78 | 75 |  | 155 | 158 |
| Median (Q1, Q3) | 32.3 (26.5, 38.9) | 33.8 (27.4, 40.7) |  | 34.1 (28.9, 42.6) | 32.6 (26.3, 39.0) |
|  |  |  |  |  |  |
| Serum Creatinine (mg/dL) |  |  |  |  |  |
| N | 78 | 71 |  | 150 | 153 |
| Median (Q1, Q3) | 1.1 (0.9, 1.5) | 1.1 (0.9, 1.4) |  | 1.4 (1.1, 1.6) | 1.2 (1.0, 1.6) |
|  |  |  |  |  |  |
| eGFR (mL/min/1.73m^2^) |  |  |  |  |  |
| N | 77 | 71 |  | 149 | 153 |
| Median (Q1, Q3) | 53.6 (38.1, 69.2) | 54.7 (41.7, 71.6) |  | 44.7 (35.0, 56.6) | 48.6 (37.9, 62.0) |
|  |  |  |  |  |  |
| Serum Potassium (mmol/L) |  |  |  |  |  |
| N | 77 | 71 |  | 149 | 151 |
| Median (Q1, Q3) | 4.3 (4.1, 4.5) | 4.3 (4.0, 4.6) |  | 4.3 (3.9, 4.6) | 4.2 (3.9, 4.5) |
|  |  |  |  |  |  |
| **Medications (Prior and Concomitant)** |  |  |  |  |  |
| Prior Use of ACEi or ARB | 45/78 (57.7%) | 37/75 (49.3%) |  | 79/155 (51.0%) | 88/158 (55.7%) |
|  |  |  |  |  |  |
| ACEi or ARB at Screening | 60/78 (76.9%) | 57/75 (76.0%) |  | 117/155 (75.5%) | 125/158 (79.1%) |
|  |  |  |  |  |  |
| MRA at Randomization | 19/78 (24.4%) | 18/75 (24.0%) |  | 56/155 (36.1%) | 42/158 (26.6%) |
|  |  |  |  |  |  |
| Beta-blocker at Randomization | 59/78 (75.6%) | 54/75 (72.0%) |  | 126/155 (81.3%) | 115/158 (72.8%) |
|  |  |  |  |  |  |
| SGLT2i at Randomization | 9/78 (11.5%) | 7/75 (9.3%) |  | 18/155 (11.6%) | 22/158 (13.9%) |
|  |  |  |  |  |  |
| Diuretic Agent at Baseline | 78/78 (100.0%) | 75/75 (100.0%) |  | 154/155 (99.4%) | 158/158 (100.0%) |
|  |  |  |  |  |  |
| **Other Characteristics** |  |  |  |  |  |
| Randomization Location |  |  |  |  |  |
| In-hospital | 53/78 (67.9%) | 56/75 (74.7%) |  | 109/155 (70.3%) | 106/158 (67.1%) |
| Out-of-hospital | 25/78 (32.1%) | 19/75 (25.3%) |  | 46/155 (29.7%) | 52/158 (32.9%) |
| Abbreviations: ACEi: angiotensin converting enzyme inhibitor; ARB: angiotensin receptor blocker; MRA: Mineralocorticoid receptor antagonist; BMI: Body Mass Index; eGFR: estimated glomerular filtration rate; BNP: B-type natriuretic peptide HF: Heart Failure; LVEF: left ventricular ejection fraction; NT-proBNP: N-terminal pro-brain natriuretic peptide; NYHA: New York Heart Association; SD: Standard Deviation; SGLT2i: Sodium-glucose Cotransporter-2 inhibitor. | | | | | |

**Supplemental Table 2 Dose Level Achieved According to Baseline Heart Failure Group and Treatment Arm**

|  | ***De novo* Heart Failure (N=153)** | |  | **Chronic Heart Failure (N=313)** | |  |
| --- | --- | --- | --- | --- | --- | --- |
| **Timepoint**  **Dose Level^1^** | **Sacubitril/Valsartan (N=78)** | **Valsartan (N=75)** | **P-value** | **Sacubitril/Valsartan (N=155)** | **Valsartan (N=158)** | **P-value** |
| Randomization (Initial Dose) |  |  |  |  |  |  |
| No. Subjects who Completed Visit | 78 | 75 |  | 155 | 158 |  |
| Assigned Dose Level |  |  | 0.785 |  |  | 0.310 |
| Dose Level 1 | 61/78 (78.2%) | 60/75 (80.0%) |  | 134/155 (86.5%) | 130/158 (82.3%) |  |
| Dose Level 2 | 17/78 (21.8%) | 15/75 (20.0%) |  | 21/155 (13.5%) | 28/158 (17.7%) |  |
| Dose Level 3 | 0/78 (0.0%) | 0/75 (0.0%) |  | 0/155 (0.0%) | 0/158 (0.0%) |  |
| No Treatment | 0/78 (0.0%) | 0/75 (0.0%) |  | 0/155 (0.0%) | 0/158 (0.0%) |  |
| Off Treatment^2^ | 0/78 (0.0%) | 0/75 (0.0%) |  | 0/155 (0.0%) | 0/158 (0.0%) |  |
|  |  |  |  |  |  |  |
| Week 4 |  |  |  |  |  |  |
| No. Subjects who Completed Visit | 72 | 72 |  | 144 | 152 |  |
| Assigned Dose Level |  |  | 0.105 |  |  | 0.183 |
| Dose Level 1 | 22/72 (30.6%) | 17/72 (23.6%) |  | 55/144 (38.2%) | 68/152 (44.7%) |  |
| Dose Level 2 | 27/72 (37.5%) | 18/72 (25.0%) |  | 35/144 (24.3%) | 37/152 (24.3%) |  |
| Dose Level 3 | 18/72 (25.0%) | 28/72 (38.9%) |  | 30/144 (20.8%) | 31/152 (20.4%) |  |
| No Treatment | 2/72 (2.8%) | 7/72 (9.7%) |  | 20/144 (13.9%) | 9/152 (5.9%) |  |
| Off Treatment^2^ | 3/72 (4.2%) | 2/72 (2.8%) |  | 4/144 (2.8%) | 7/152 (4.6%) |  |
|  |  |  |  |  |  |  |
| Week 8 |  |  |  |  |  |  |
| No. Subjects who Completed Visit | 64 | 63 |  | 132 | 130 |  |
| Assigned Dose Level |  |  | 0.409 |  |  | 0.092 |
| Dose Level 1 | 15/64 (23.4%) | 12/63 (19.0%) |  | 32/132 (24.2%) | 49/130 (37.7%) |  |
| Dose Level 2 | 14/64 (21.9%) | 11/63 (17.5%) |  | 23/132 (17.4%) | 24/130 (18.5%) |  |
| Dose Level 3 | 28/64 (43.8%) | 31/63 (49.2%) |  | 42/132 (31.8%) | 37/130 (28.5%) |  |
| No Treatment | 5/64 (7.8%) | 9/63 (14.3%) |  | 25/132 (18.9%) | 14/130 (10.8%) |  |
| Off Treatment^2^ | 2/64 (3.1%) | 0/63 (0.0%) |  | 10/132 (7.6%) | 6/130 (4.6%) |  |
| ^1^Dose Level 1: 40 mg Valsartan or 24/26 mg LCZ696, BID; Dose Level 2: 80 mg Valsartan or 49/51 mg LCZ696, BID; Dose Level 3: 160 mg Valsartan or 97/103 mg LCZ696, BID. ^2^Subjects who prematurely discontinued study treatment but continued with visits. | | | | | | |
